# Supplementary figures and images for: Effect of Irrigation on Sugarcane Morphophysiology in the Brazilian Cerrado
Source: Plants (Basel). 2024 Mar 23;13(7):937. doi: 10.3390/plants13070937 (PMC11013474; doi:10.3390/plants13070937)

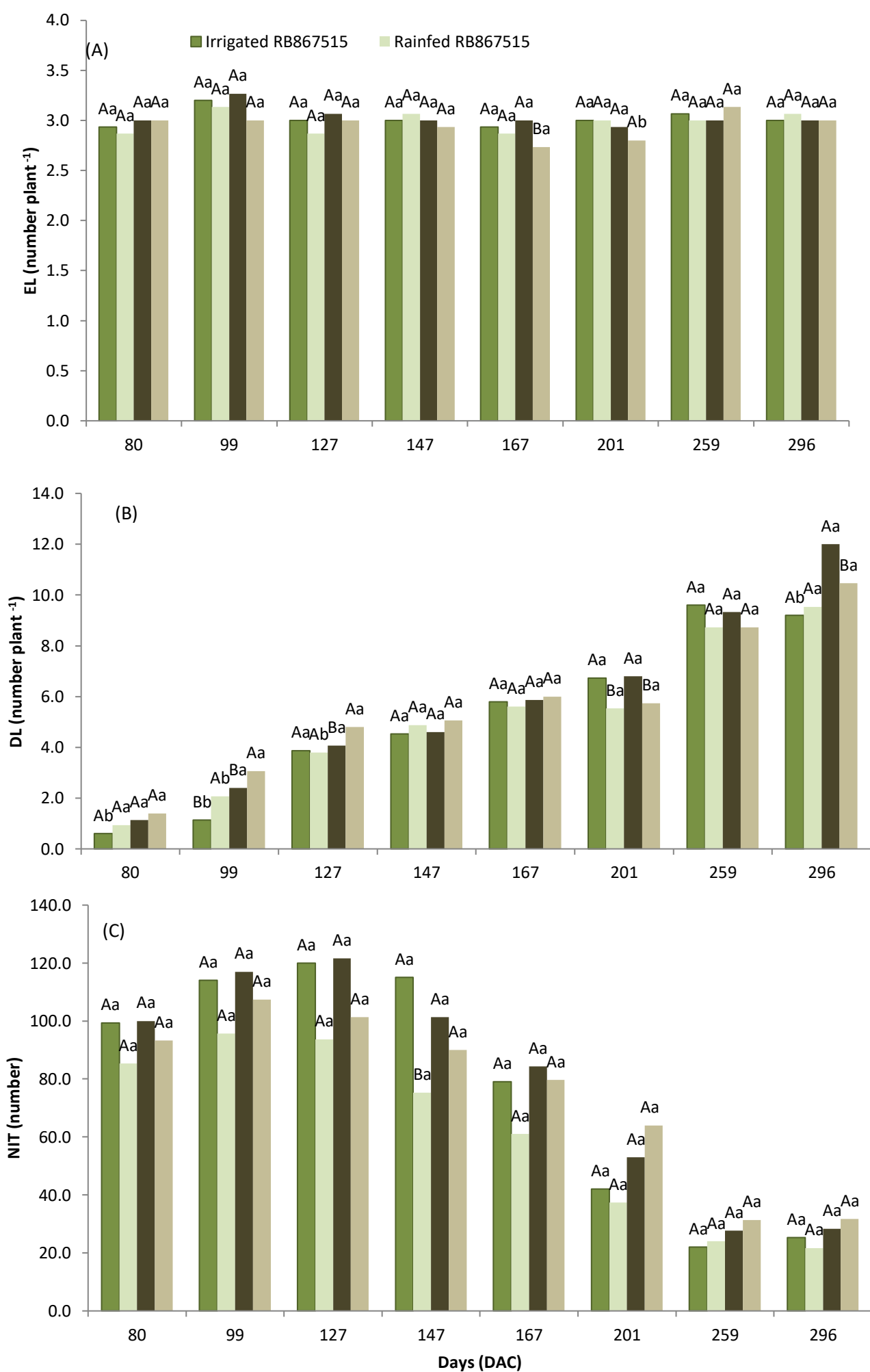

Supplement: Supplementary file 1 [file plants-13-00937-s001.zip › plants-2889540-supplementary.pdf]
